# Evolution of Endoscopic and Histologic Assessment of Eosinophilic Esophagitis in Europe and Evolution of the Diagnostic Delay: Data From the EUREOS EoE CONNECT Registry

**DOI:** 10.1002/ueg2.70271

**Published:** 2026-08-12

**Authors:** E. Grueso‐Navarro, E. Dilaghi, J. Fernández‐Pacheco, E. Amorena, E. V. Savarino, L. Blas‐Jhon, I. Pérez‐Martínez, A. Granja‐Navacerrada, D. Guagnozzi, L. Martín‐Asenjo, G. Pellegatta, M. Coletta, C. García‐Suárez, S. de la Riva, L. de la Peña‐Negro, E. Betoré, A. L. Krarup, J. Barrio, M. Votto, L. Bujanda, S. Fernández‐Fernández, J. E. Naves, A. González‐Almeida, R. Honrubia‐López, S. Carrión, S. Oliva, C. Caño‐Cerdán, M. L. Masiques‐Mas, S. Espina‐Cadena, J. González‐Cervera, S. Casabona‐Francés, J. Nicolay‐Maneru, V. Úbeda‐Vargas, M. Ghisa, P. Romo‐de Arriba, C. Donado, A. Benagues, V. Martín‐Domínguez, O. Nantes‐Castillejo, L. Arias‐González, D. Maniero, H. Moreira‐González, I. Maray, J. Barrio‐Torres, M. T. Pérez‐Fernández, M. Ostiz‐Llanos, L. Rodríguez‐Alcolado, A. M. Cabrera‐Martínez, E. J. Laserna‐Mendieta, C. Santander, A. J. Lucendo

**Affiliations:** ^1^ Department of Gastroenterology Hospital General de Tomelloso Tomelloso Spain; ^2^ Instituto de Investigación Sanitaria de Castilla‐La Mancha (IDISCAM) Tomelloso Spain; ^3^ Centro de Investigación Biomédica en Red Enfermedades Hepáticas y Digestivas (CIBERehd) Madrid Spain; ^4^ Department of Medical‐Surgical Sciences and Translational Medicine Sant'Andrea Hospital Sapienza University of Rome Rome Italy; ^5^ Instituto de Investigación Sanitaria la Princesa Madrid Spain; ^6^ Department of Gastroenterology, Hospital Universitario de la Princesa Madrid Spain; ^7^ Department of Gastroenterology Hospital Universitario de Navarra Pamplona Spain; ^8^ Surgery, Oncology and Gastroenterology Gastroenterology Unit, Azienza Ospedaliera di Padova Padova Italy; ^9^ Department of Gastroenterology Fundación Jiménez Díaz Madrid Spain; ^10^ Department of Pharmacy Hospital Universitario Central de Asturias Oviedo Spain; ^11^ Diet Microbiota and Healh Group Instituto de Investigación Sanitaria del Principado de Asturias (ISPA) Oviedo Spain; ^12^ Department of Pediatric Gastroenterology Hospital Universitario de Fuenlabrada Fuenlabrada Spain; ^13^ Department of Gastroenterology Hospital Universitario Vall d’Hebrón Barcelona Spain; ^14^ Department of Gastroenterology Hospital Universitario de Álava Vitoria Spain; ^15^ Endoscopy Unit Department of Gastroenterology IRCCS Humanitas Research Hospital Rozzano Italy; ^16^ Department of Gastroenterology and Endoscopy Fondazione IRCCS Ca’ Granda Ospedale Maggiore Policlinico Milan Italy; ^17^ Department of Gastroenterology Hospital Universitario de Cabueñes Gijón Spain; ^18^ Department of Gastroenterology Clínica Universitaria de Navarra Pamplona Spain; ^19^ Department of Gastroenterology Hospital de Viladecans Barcelona Spain; ^20^ Department of Gastroenterology Hospital Universitario Miguel Servet Zaragoza Spain; ^21^ Department of Emergency Medicine and Trauma Center and Gastroenterology Aalborg University Hospital Aalborg Denmark; ^22^ Department of Clinical Medicine Aalborg University Aalborg Denmark; ^23^ Department of Gastroenterology Hospital Universitario Rio Hortega Valladolid Spain; ^24^ Pediatric Unit Department of Clinical Surgical Diagnostic and Pediatric Sciences University of Pavia Pavia Italy; ^25^ Department of Gastroenterology Biogipuzkoa Health Research Institute Universidad del Pais Vasco (UPV/EHU) San Sebastián Spain; ^26^ Department of Pediatric Gastroenterology Hospital Universitario Severo Ochoa Leganés Spain; ^27^ Department of Gastroenterology Parc de Salut Mar Barcelona Spain; ^28^ Department of Gastroenterology Complejo Hospitalario Universitario Insular Materno Infantil Las Palmas de Gran Canaria Spain; ^29^ Department of Gastroenterology Hospital Universitario Infanta Sofía San Sebastián de los Reyes Spain; ^30^ Department of Medicine Universidad Europea de Madrid Faculty of Biomedical and Health Sciences Madrid Spain; ^31^ Department of Gastroenterology Hospital Universitario de Mataró Mataró Spain; ^32^ Department of Pediatric Digestive Endoscopy University Hospital Umberto I & Sapienza University of Rome Rome Italy; ^33^ Department of Gastroenterology Hospital de Santa Bárbara Soria Spain; ^34^ Department of Pediatric Gastroenterology Hospital de Granollers Granollers Spain; ^35^ Department of Gastroenterology Hospital General de la Defensa Zaragoza Spain; ^36^ Department of Allergy Hospital General Universitario de Toledo Toledo Spain; ^37^ Department of Allergy Hospital Universitario Central de Asturias Oviedo Spain; ^38^ Department of Surgery Medical and Social Sciences Universidad de Alcalá Alcalá de Henares Spain

**Keywords:** biopsy, diagnosis, diagnostic delay, endoscopy, Eosinophilic esophagitis, guidelines, registry, temporal trends

## Abstract

**Background:**

Endoscopy with histopathological assessment of esophageal biopsies is the cornerstone for the diagnosis and phenotypic characterization of eosinophilic esophagitis (EoE).

**Objective:**

To evaluate temporal trends in endoscopic assessment and biopsy sampling practices for EoE in a large European cohort and their association with temporal trends in diagnostic delay (DD).

**Methods:**

A cross‐sectional analysis was conducted using multicenter data from the EoE CONNECT registry. Endoscopic findings and biopsy data at diagnosis were analyzed. Temporal trends in biopsy strategies, EREFS implementation, and DD were assessed. Diagnostic sensitivity of individual esophageal segments and their combinations was evaluated in patients undergoing multi‐segment sampling. Associations between guideline publication and biopsy practices were analyzed using logistic regression models.

**Results:**

A total of 3298 patients were included. Biopsies were obtained from one esophageal segment in 12.6% of patients, two segments in 63.6%, and three segments in 23.8%. Adherence to guideline‐recommended biopsy strategies improved over time, with reduced single‐segment sampling and increased use of ≥ 2 segments. Distal esophageal biopsies provided the highest diagnostic sensitivity, and sampling that included the distal segment achieved sensitivity above 97%, particularly when combined with middle esophageal biopsies. EREFS reporting increased from 88–90% to over 98% in recent years. DD decreased significantly over time (approximately 0.7 years per calendar year), accompanied by a shift from mixed/stricturing to inflammatory phenotypes.

**Conclusion:**

Increased alignment with international guidelines, reflected by standardized endoscopic reporting and multi‐segment biopsy protocols, was associated with improved diagnostic sensitivity, reduced DD, and fewer fibrostricturing presentations.

## Key Summary

- Summarise the established knowledge on this subject
  ◦ Eosinophilic esophagitis (EoE) is a chronic disease characterized by esophagus dysfunction and eosinophil‐predominant inflammation of the esophageal mucosa, which is patchily distributed along the organ.
  ◦ Patients with EoE experience prolonged diagnostic delays across all reported countries, and published trends of small studies suggest that adherence to clinical guidelines may reduce the time to diagnosis.
  ◦ Data on the extent to which real‐world EoE diagnostic practices adhere to clinical practice guideline recommendations, and on their impact on patient outcomes over time, remain limited.
- What are the significant and/or new findings of this study?
  ◦ Compliance with guideline‐recommended biopsy strategies increased over time, with reduced reliance on single‐segment endoscopic sampling and greater use of biopsies from ≥ 2 esophageal segments following the 2017 guideline publication.
  ◦ Distal esophageal biopsies yielded the highest diagnostic sensitivity; sampling protocols that included the distal segment achieved sensitivity exceeding 97%, particularly when combined with mid‐esophageal biopsies.
  ◦ Standardized reporting of endoscopic findings using the EREFS classification increased from 88% to over 98% in recent years.
  ◦ Diagnostic delay in EoE decreased significantly over time (by approximately 0.7 years per calendar year), resulting in fewer patients presenting with mixed or fibrostricturing phenotypes at diagnosis, and a corresponding increase in inflammatory presentations.

## Graphical abstract

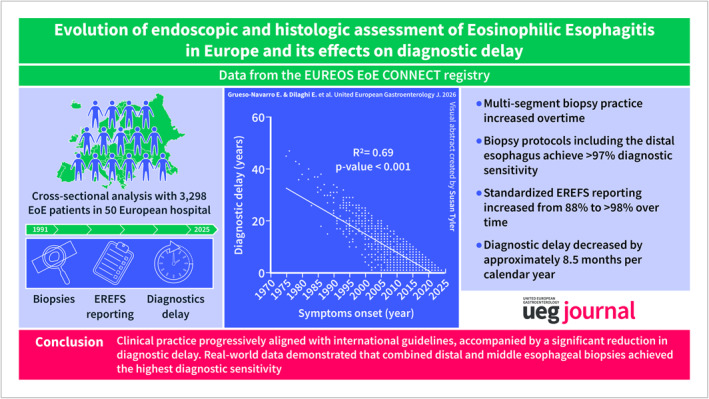

**Abbreviations**
CI: confidence interval
DD: diagnostic delay
DSS: Dysphagia Symptom Score
EoE: eosinophilic esophagitis
eos/hpf: eosinophils per high‐power field
EREFS: edema, rings, exudates, furrows, strictures
GERD: gastroesophageal reflux disease
OR: Odds ratio
PEC: peak eosinophil count
PPI: proton pump inhibitors

## 1 Introduction

Eosinophilic esophagitis (EoE) is a chronic, immune‐mediated disease defined by symptoms of esophageal dysfunction and dense eosinophil infiltration in the esophageal mucosa. [1] Its global epidemiology has expanded rapidly, [2] and it has transitioned from a rare disease to a common disorder, with a ninefold increase in prevalence. [3] In fact, EoE represents the most common cause of dysphagia among children and young adults, and is the second cause of chronic dysphagia after gastroesophageal reflux disease (GERD). [2] Since EoE was first described in the early 1990s, [4, 5] diagnostic approaches have evolved considerably. Early definitions emphasized mucosal eosinophil infiltration ranging between 5–30 eosinophils per high‐power field (eos/HPF), [6] until the first consensus guidelines in 2007 [7] formally established the ≥ 15 eos/hpf threshold. Recognition of the patchy distribution of eosinophilic infiltration along the esophagus [8] supported the adoption of multi‐site biopsies. [9]

Historically, proton pump inhibitors (PPIs) were used diagnostically to exclude GERD, [10] but since the 2017 international guidelines [1] they have been redefined as a first‐line therapeutic option [11]. Because EoE presents with heterogeneous symptoms that limit clinical diagnostic accuracy, [12, 13] recent guidelines have emphasized a multimodal diagnostic strategy integrating clinical, histologic and endoscopic criteria, as endoscopic features were recognized as biologically relevant. [14] Accordingly, current recommendations [1, 14, 15] advocate the use of standardized endoscopic reporting systems such as EREFS, [16] and define EoE by the combination of dysphagia symptoms with histologic evidence of ≥ 15 eos/hpf in esophageal mucosa, obtained from at least six biopsies across a minimum of two segments along the esophagus.

Over almost two decades, multiple international guidelines, [1, 14, 15, 17] national guidelines, [18, 19, 20] consensus statements, [7, 21, 22, 23, 24, 25] and technical reviews [26, 27] have structured the basis for EoE diagnosis and management. Although diagnosis of EoE has improved over time in Europe, [28, 29] other works highlight irregular adherence to these recommendations, [30, 31, 32] resulting in underdiagnosis with a parallel influence on diagnostic delay (DD). [33, 34] Observational studies describing real‐world diagnostic practices in EoE are scarce. Therefore, in this study, we sought to describe temporal trends in endoscopic assessment and biopsy practices in a large cohort of European EoE patients and to evaluate changes in DD throughout the study period.

## 2 Material and Methods

### 2.1 Study Design and Dataset

Here we performed a cross‐sectional analysis of multicenter, prospectively‐collected data at EoE CONNECT, the European registry for EoE supported by EUREOS (European Consortium for Eosinophilic Diseases of the GI Tract). EoE CONNECT constitutes a large, clinically reliable source of EoE management‐related clinical data with public registering protocols and definitions. [35]

**FIGURE 1:**
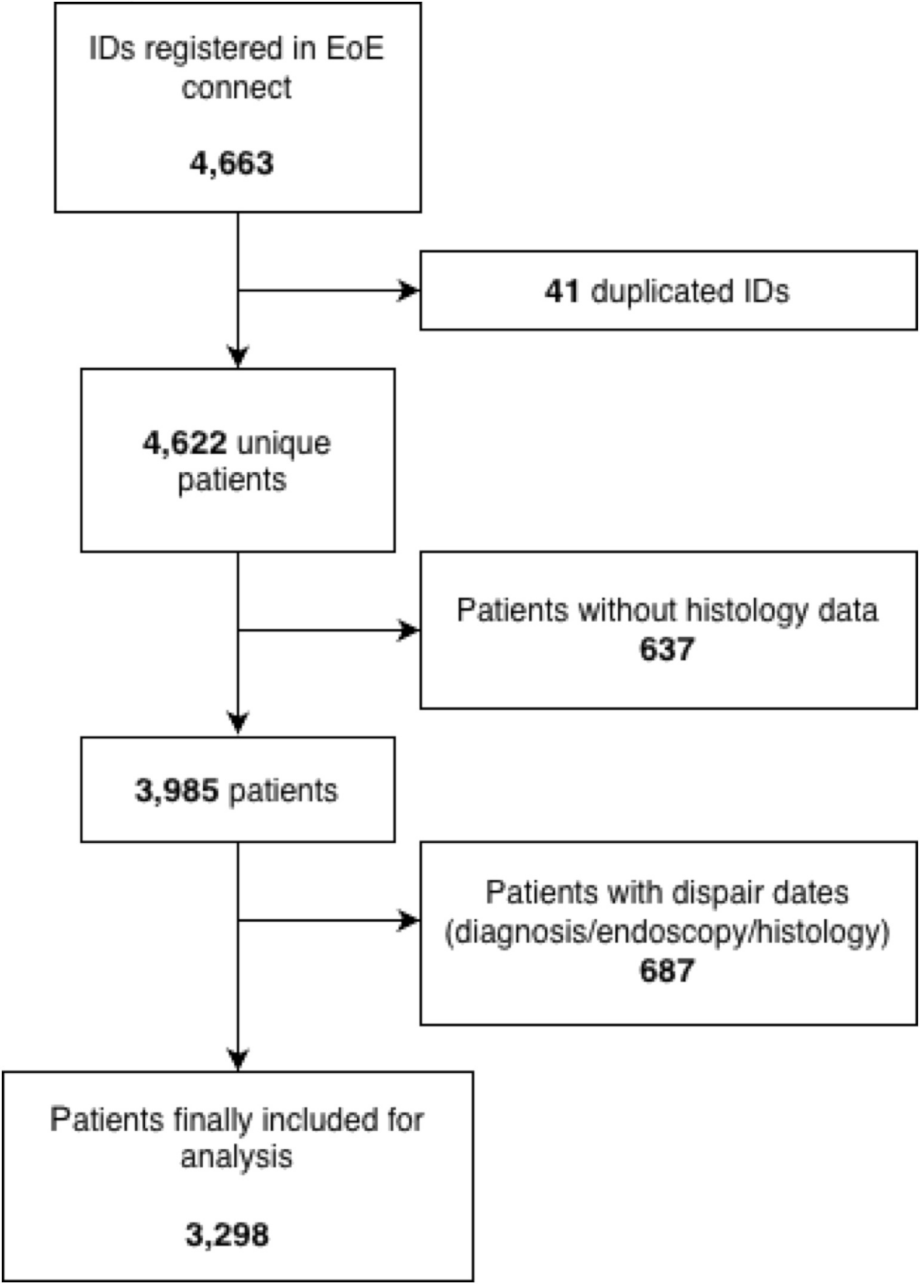
Patient selection flowchart for the EoE CONNECT registry analysis.

On October 6th, 2025, 4663 patient IDs were retrieved from the EoE CONNECT registry. After applying predefined eligibility criteria (Figure 1, Supporting Information S1), 3298 patients were included.

To evaluate changes in clinical practice and adherence to diagnostic standards, the 2017 guidelines [1] were used as the reference, as they represent the first multi‐society, evidence‐based document developed using the GRADE methodology. This article was drafted according to STROBE guidelines for observational studies to ensure the quality of reporting [36].

### 2.2 Variables and Definitions

The following variables were analyzed: age at diagnosis, sex, country and hospital of diagnosis, year of: symptom onset, diagnosis, endoscopy and histology reports; symptoms at diagnosis, Dysphagia Symptom Score (DSS), overall EREFS score and components (Edema, Rings, Exudates, Furrows and Stenosis), esophageal segment biopsied for diagnosis (proximal, middle and distal esophagus) and histology results. Definitions of hospital caseload, diagnostic delay, peak eosinophil count, pathologic biopsy and EoE phenotype are detailed in Supporting Information S1.

### 2.3 Statistical Analysis

Continuous and categorical variables were compared with non‐parametric and chi‐square tests as appropriate. Temporal trends were analyzed using linear regression, and the association between guideline‐defined periods and multi‐segment biopsy sampling using logistic regression models. Detailed statistical methods are provided in Supporting Information S1. Statistical significance was set at *p* < 0.05; analyses were performed in SPSS v25.0 and GraphPad Prism v9.5.1.

### 2.4 Ethical Considerations

The study adhered to the principles outlined in the Helsinki Declaration, which sets ethical guidelines for human‐related research. EoE CONNECT obtained approval from the Ethics Research Committee of Hospital Universitario de La Princesa (Madrid, Spain) as Central committee and from the corresponding committee in each participating center. Written informed consent from all patients or their legal guardians was obtained.

## 3 Results

### 3.1 Study Cohort and Baseline Characteristics

A total of 3298 patients with confirmed EoE were included after application of predefined eligibility criteria (Figure 1). These patients were enrolled from 50 hospitals across four European countries participating in the EoE CONNECT registry, with data recorded up to October 6th 2025. Detailed demographic and clinical characteristics are summarized in Table 1.

**TABLE 1:** Demographic and clinical characteristics of patients with eosinophilic esophagitis (EoE) patients from EoE CONNECT registry.

	EoE patients *n* = 3298
Male sex, *n* (%) *n* = 3298	2454 (74.4)
Age at diagnosis, mean (SD)	35.2 (15.2)
Adults (> 18 years old), *n* (%)	2637 (82.0)
Age distribution at diagnosis, *n* = 3216	
Children (< 12 years old), *n* (%)	226 (7.0)
Adolescent (12–17 years old), *n* (%)	353 (11.0)
Adults (18–65 years old), *n* (%)	2570 (79.9)
Elderly (> 65 years old), *n* (%)	67 (2.1)
Country of registry, *n* = 3298	
Spain, *n* (%)	2736 (83.0)
Italy, *n* (%)	514 (15.5)
Denmark, *n* (%)	42 (1.3)
France, *n* (%)	6 (0.2)
Type of hospital, *n* = 3298	
Low‐volume, *n* (%)	1190 (36.1)
High‐volume, *n* (%)	2108 (63.9)
Time of diagnosis according to 2017 guidelines	
Before 2017, *n* (%)	978 (29.7)
After 2017, *n* (%)	2320 (70.3)
EoE phenotype, *n* = 3024	
Inflammatory, *n* (%)	1862 (61.6)
Mixed‐stricturing, *n* (%)	851 (28.1)
Without EoE endoscopic features, *n* (%)	311 (10.3)
Diagnostic delay, years, median (IQR), *n* = 2810	2.0 (0–6)
Peak eos/hpf (overall), median (IQR), *n* = 2830	40 (26–60)
Peak eos/hpf (proximal esophagus), median (IQR), *n* = 2238	25 (11–45)
Peak eos/hpf (middle esophagus), median (IQR), *n* = 952	35 (20–50)
Peak eos/hpf (distal esophagus), median (IQR), *n* = 2585	35 (20–50)
Dysphagia symptom score, median (IQR), *n* = 2468	5 (0–9)
EREFS score, median (IQR) *n* = 3114	3 (2–4)
EREFS inflammatory subscore, median (IQR)	2 (1–3)
EREFS fibrotic subscore, median (IQR)	1 (0–1)

### 3.2 Biopsy Sampling Practices and Diagnostic Sensitivity Across Esophageal Segments

At diagnosis, biopsies were obtained from a single esophageal segment in 417 patients (12.6%), from two segments in 2096 (63.6%), and from all three in 785 (23.8%), with a mean of 2.11 (SD 0.59) sampled segments per patient.

**FIGURE 2:**
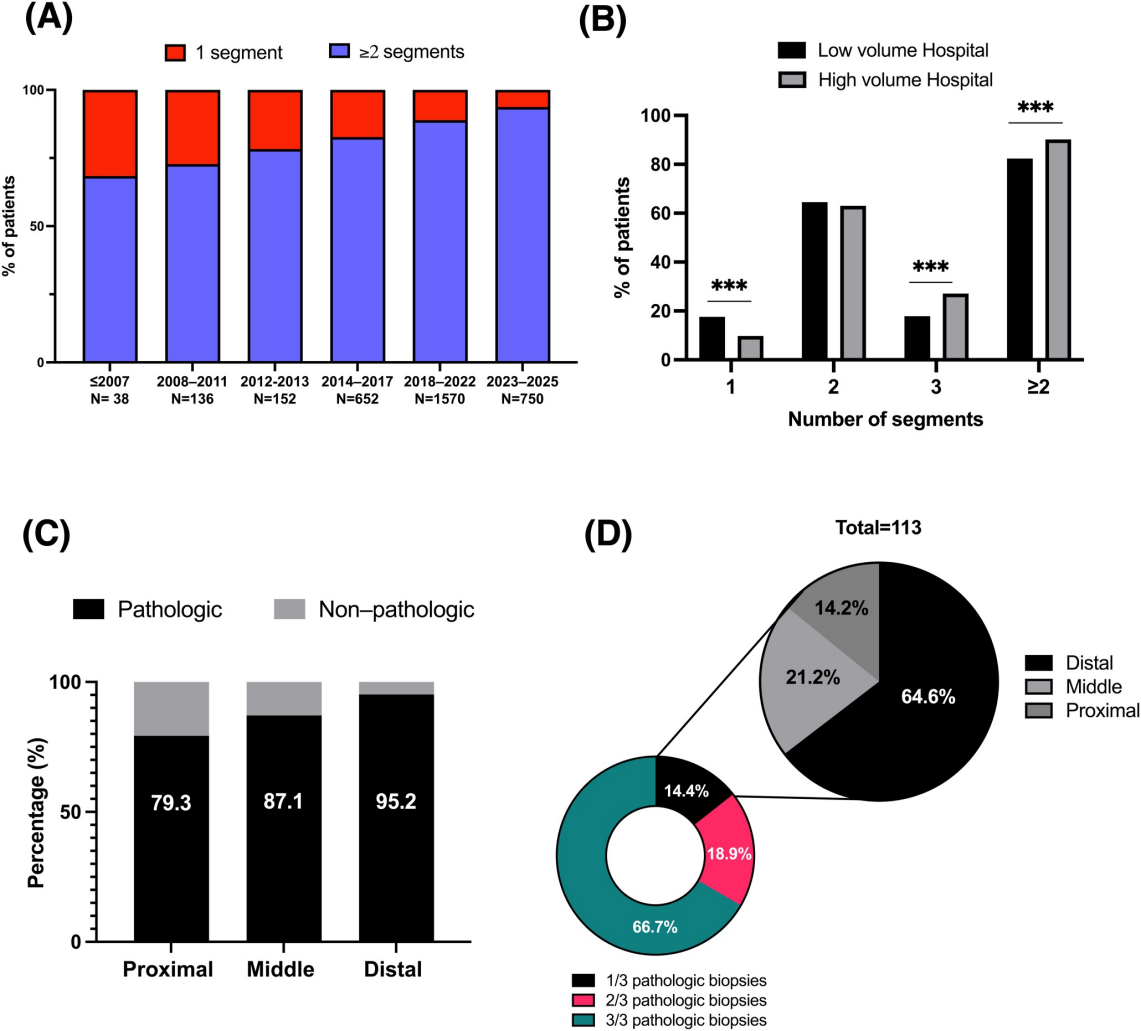
Biopsy sampling practices and segment‐specific distribution of pathologic findings. (A) Proportion of patients sampled with 1 segment versus ≥ 2 esophageal segments across the six diagnostic periods, (B) Proportion of patients sampled with 1, 2, or 3 esophageal segments stratified by hospital case volume (high vs low). (C) Stacked‐bar plot showing the percentage of pathologic and not pathologic percentage of sampled biopsies in proximal, middle or distal esophagus. (D) Pie chart showing distribution of pathologic biopsies across the three esophageal segments in the subgroup of patients with 3‐segment sampling at diagnosis (*n* = 785). ^***^ *p* < 0.001.

To assess the influence of guideline publication on sampling practices, patients were stratified into six diagnostic periods defined by major guidelines publication years (2007, [7] 2011, [22] 2013 [17], 2017 [1] and 2022 [18, 37]). The proportion of patients undergoing biopsies from ≥ 2 esophageal segments significantly differed between time periods (*p* < 0.001), increasing to more than 90.4% after 2018 (Figure 2A). The odds of multi‐segment biopsy increased across periods compared with the ≤ 2007 reference group, with statistically significant associations observed in the 2018–2022 and 2023–2025 sub‐periods, reflecting a continuous and cumulative adoption of the 2017 guideline recommendations (Table 2). A linear regression analysis confirmed a significant increase in the proportion of patients undergoing ≥ 2 segment sampling over time (B = 1.22% per year; *p* < 0.001)(Supporting Information S1: Figure S1). Sensitivity analyses repeating the same logistic regression with the 2008–2011, 2012–2013, and 2014–2017 periods as alternative reference categories yielded fully consistent results, (Supporting Information S1. Multi‐segment sampling was significantly more frequent in high‐ than in low‐volume centers (Figure 2B), and the proportion of ≥ 2‐segment biopsies increased significantly after 2017 in both groups (Table 3), reflecting broad adherence to updated guidelines.

**TABLE 2:** Association between guideline‐defined time periods and the probability of obtaining ≥ 2 esophageal biopsy segments.

	*N*	Patients undergoing ≥ 2‐segment biopsy sampling, *n* (%)	OR (95% CI)	*p*‐value
≤ 2007 (reference)	38	26 (68.4)	–	–
2008–2011	136	99 (72.8)	1.23 (0.57–2.70)	0.60
2012–2013	152	119 (78.3)	1.66 (0.76–3.65)	0.20
2014–2017	652	539 (82.7)	2.20 (1.08–4.50)	0.030
2018–2022	1570	1394 (88.7)	3.68 (1.82–7.42)	< 0.001
2023–2025	750	703 (93.7)	6.90 (3.28–14.54)	< 0.001

*Note:* Binary logistic regression model using the period ≤ 2007 as the reference category.

Abbreviations: CI: confidence intervals; N: total number of patients diagnosed within each time period; OR: Odds ratio.

**TABLE 3:** Differences in number of biopsy segments, report of EREFS and distribution of EoE phenotypes based on 2017 international guidelines according to year of diagnosis and the hospital case volume.

	Before 2017	After 2017	Absolute difference (%)	*p*‐value
Low‐volume hospitals				
≥ 2 segments, *n* (%)	232 (73.7)	748 (85.5)	+11.8	< 0.001
EREFS provided, *n* (%)	288 (91.4)	849 (97.0)	+5.6	< 0.001
Inflammatory phenotype, *n* (%)	143 (53.2)	509 (61.7)	+8.5	0.01
Mixed‐stricturing phenotype, *n* (%)	85 (31.6)	220 (26.7)	−4.9	0.12
High‐volume hospitals				
≥ 2 segments, *n* (%)	551 (83.1)	1350 (93.4)	+10.3	< 0.001
EREFS provided, *n* (%)	579 (87.3)	1398 (96.7)	+9.4	< 0.001
Inflammatory phenotype, *n* (%)	305 (54.6)	905 (66.0)	+11.4	< 0.001
Mixed‐stricturing phenotype, *n* (%)	186 (33.3)	360 (26.3)	−7.0	0.002

Across all centres, distal biopsies were the most frequently sampled site and yielded the highest proportion of pathologic findings, confirming a distal‐to‐proximal gradient of disease activity (Figure 2C). This was validated in the subgroup of patients with three‐segment sampling (n = 785) (Figure S2). Since multi‐segment biopsy is convenient, we evaluated the frequencies of segment combinations. Proximal + distal sampling was the most common combination (86.7%), followed by distal + middle (11.4%) and proximal + middle (1.9%). In patients undergoing three‐segment sampling (*n* = 785), 66.7% showed above‐threshold eosinophilia (≥ 15 eos/hpf) at all sites. Among those with isolated eosinophilia in one out of the three segments (14.4%), the distal site was most informative (64.6% of pathologic biopsies), and the proximal least (14.2% of pathologic biopsies) (Figure 2D). When patients were dichotomized into pediatric and adult groups, pediatric patients showed a higher frequency of proximal esophageal involvement compared with adults (81.1% vs. 72.4%; *p* = 0.02). No differences in segment‐specific pathologic findings were observed according to sex.

Then, we evaluated the diagnostic sensitivity of different 2‐segment combinations in the three‐segment sampling group (*n* = 785). Distal + middle sampling achieved the highest sensitivity for EoE diagnosis (97.9%, 95% CI 96.97–98.96%), followed by distal + proximal (97.0%, 95% CI 95.74–98.15%) and middle + proximal segments (90.7%, 95% CI 88.67–92.73%). Differences in diagnostic sensitivity between 2‐segment combinations were explored by pairwise analysis across strategies. When middle + proximal was compared with either distal + middle or distal + proximal combinations, differences were statistically significant (*p* < 0.001), but no when both combinations included the distal segment (distal + middle vs. distal + proximal; *p* = 0.27).

### 3.3 Temporal Trends in EREFS Implementation and Endoscopic Phenotypes

**FIGURE 3:**
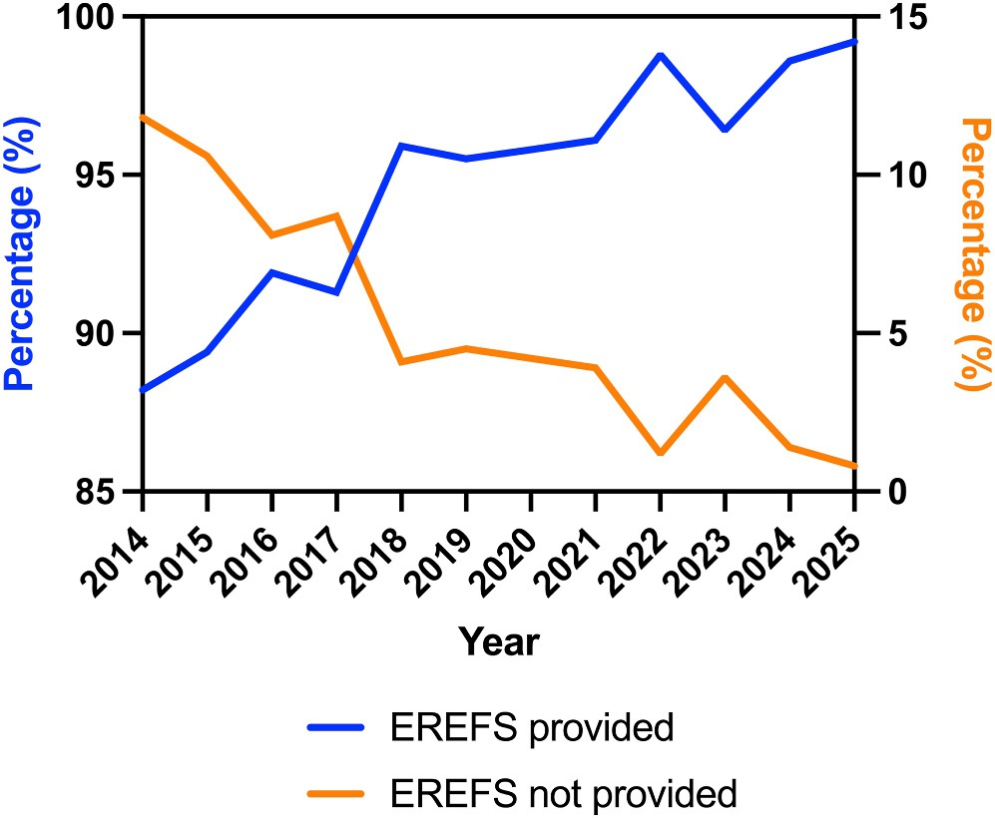
Evolution of EREFS reporting in endoscopic characterization of EoE over the years. The proportion of patients with a documented EREFS score is shown per calendar year.

The analysis of EREFS score implementation included data from 2014 onward, as the EREFS classification was published in 2013. [16] Over the study period, data showed a clear and progressive improvement in EREFS score reported across participating centers. In the earliest years of the registry, ∼10% of patients lacked a documented EREFS score, but this percentage steadily decreased to less than 1% by 2025 (Figure 3). This downward trend persisted following the publication of the 2017 European EoE guidelines, regardless of the hospital category (Table 3).

### 3.4 Diagnostic Delay Trends Over Time

**FIGURE 4:**
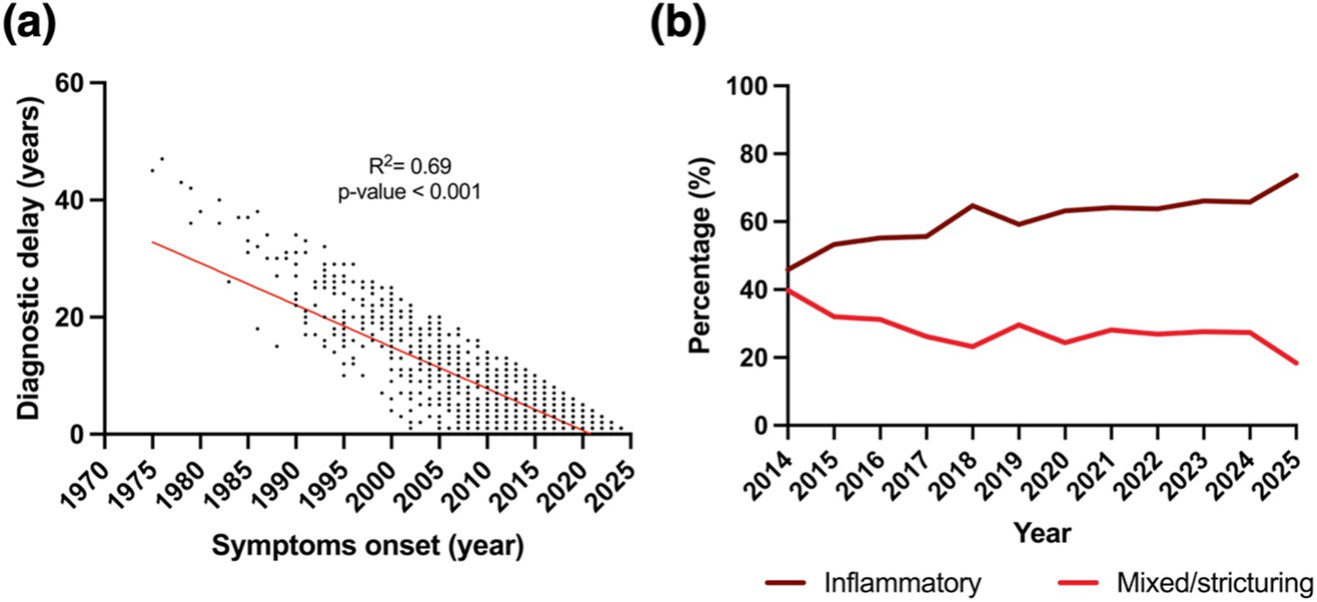
Diagnostic delay (DD) trends and endoscopic phenotype evolution over time. (A) Linear regression of DD on year of symptom onset in the overall population (*n* = 2810). (B) Distribution (%) of inflammatory and mixed/stricturing phenotypes per calendar year from 2014 onward. The number of patients diagnosed each year was: 2014, *n* = 119; 2015, *n* = 142; 2016, *n* = 173; 2017, *n* = 218; 2018, *n* = 295; 2019, *n* = 336; 2020, *n* = 261; 2021, *n* = 356; 2022, *n* = 322; 2023, *n* = 335; 2024, *n* = 289; 2025, *n* = 126. The reduction in 2020 reflects the temporary decline in endoscopic activity associated with the COVID‐19 pandemic. Data for 2025 correspond to a partial calendar year, with diagnoses recorded up to October 6, 2025 (the cut‐off for data extraction).

The analysis of DD included all patients with available data on the year of symptom onset (*n* = 2810). Linear regression analysis revealed a statistically significant inverse association between the year of symptom onset and DD (*p* < 0.001), with DD decreasing by approximately 8.5 months per calendar year (Figure 4A). This temporal trend paralleled a progressive decline in mixed/stricturing phenotypes and a corresponding increase in inflammatory presentations (Figure 4B). A consistent temporal trend was observed in both low‐ and high‐volume hospitals after 2017 (Table 3). The increase in inflammatory phenotype was statistically significant in both settings, whereas a significant reduction in the mixed/stricturing phenotype was observed only in high‐volume hospitals (*p* = 0.002). These findings suggest a more pronounced association of reduced DD on phenotype distribution in high‐volume centers.

Independent analysis of DD by phenotype revealed a significant decrease for both groups (*p* < 0.001), with a steeper reduction in the mixed/stricturing phenotype (9.4 months/year) than in the inflammatory one (8.0 months/year).

## 4 Discussion

This study provides the most comprehensive evaluation of temporal changes in endoscopic assessment and biopsy sampling at EoE diagnosis, showing improved guideline adherence and diagnostic performance of biopsy strategies. These advances were paralleled by reduced DD, particularly in mixed/stricturing phenotypes, reflecting the growing integration of evidence‐based practices across Europe. The 2017 European guidelines were used as the reference, as they were the first multi‐society, evidence‐graded document defining standardized diagnostic criteria. [1] Subsequent publications reinforced multi‐segment sampling and standardized EREFS reporting without changing the core 2017 diagnostic recommendations. [18, 37] Accordingly, improved biopsy practices after 2017 reflect cumulative guideline adoption. The updated 2025 American guidelines [14] were not considered, as their recent publication precluded meaningful influence during the study period. The only national EoE guidelines available among participating countries, published in Italy [24] and France [25], reiterate the same diagnostic recommendations established in 2017 without introducing a novel approach.

The progressive increase in the number of esophageal segments sampled over time, especially after major international guidelines, reflects growing awareness of the need for tissue sampling to improve diagnostic reliability. This evolution aligns with the inherently patchy distribution of eosinophilic inflammation in EoE [8, 38] where inflamed areas often lie adjacent to histologically normal mucosa, so single‐site sampling may yield false negatives and underestimate disease burden. Recent studies in adult [39, 40] and pediatric cohorts [41] confirmed this heterogeneity showing discordant histologic results along the esophagus, agreeing in a denser eosinophilia and more common pathologic findings in the distal segment compared to middle and proximal locations. Therefore, multi‐segment biopsy protocols are pivotal to achieve accurate histologic diagnosis of EoE. Since the first consensus document [38], and subsequent guidelines [1, 14, 21, 22] sampling of distal and proximal esophagus were recommended to maximize diagnosis. However, evidence supporting optimal location for biopsy sampling remains limited, particularly from studies evaluating patients with three‐segment sampling at diagnosis, thereby minimizing bias due to missing segment data. Our study comprising 785 of such patients, considerably expands previous works that addressed this question with over 60 patients [39, 41] and a third, more recent, with a larger cohort (263 patients) but largely composed of patients in clinical remission or post‐treatment, limiting direct comparability with diagnostic‐stage disease [42]. Nonetheless, previous and current data agree on a distal‐to‐proximal sensitivity gradient. Distal samples most frequently showed histologic activity, with progressively lower positivity rates in middle and proximal segments. In our cohort, sensitivity reached 91.8% in the distal, 85.5% in the middle, and 74.9% in the proximal esophagus. For biopsy combinations, inclusion of the distal segment yielded sensitivity above 97%, whereas the proximal + middle combination was considerably lower (90.7%). Nevertheless, three‐segment sampling still provided an incremental diagnostic benefit of about 2%, according to the prevalence of isolated proximal eosinophilic involvement. These findings support proximal biopsies and comprehensive three‐segment sampling, particularly in pediatric patients, in whom proximal involvement was significantly more frequent. A prospective pediatric cohort similarly showed that combining distal biopsies with either middle or proximal sampling achieves ∼98% diagnostic yield, whereas mid‐proximal combinations remain suboptimal [41]. Taken together, these findings indicate that distal sampling is recommended for optimal diagnostic yield, and the choice between middle or proximal as the second segment is diagnostically equivalent and may be guided by the presence of endoscopic features or practical considerations. However, proximal sampling captures the small proportion of patients with isolated proximal disease and is particularly relevant in pediatric patients, in whom proximal involvement was more frequent than in adults in our cohort (81.1% vs 72.4%; *p* = 0.02). Therefore, three‐segment sampling remains the preferred standard whenever clinically feasible. This may be especially important in children and adolescents, in whom the greater frequency of proximal involvement could increase the risk of missed diagnoses when proximal biopsies are omitted.

EREFS implementation across EoE CONNECT centers reflected improved standardization of endoscopic assessment. Since its introduction in 2013 [43], EREFS scoring increased from about 88–90% to more than 98% by 2025, while missing reports declined. This likely reflects awareness of structured reporting, reinforced by the 2017 European guidelines and subsequent consensus recommendations [21]. Both high‐ and low‐volume centers showed parallel improvements in biopsy sampling and EREFS documentation, suggesting widespread adoption of standardized protocols and a general enhancement in diagnostic quality across Europe. Likewise, diagnostic delay and trend in phenotype distribution were not influenced by hospital‐volume, indicating that temporal trends were not exclusively driven by highly experienced centers. The temporal analysis revealed a progressive reduction in DD and a marked shift in endoscopic phenotypes, with a decline in mixed/stricturing forms and an increase in inflammatory presentations. Chronic, untreated inflammation is known to drive fibrotic remodeling and luminal narrowing, a process clearly documented in previous studies showing the prevalence of strictures rose from 19% among patients diagnosed within two years of symptom onset to 52% in those with delays exceeding 20 years [16]. Similarly, a multi‐center Dutch cohort reported a 9% increase in the odds of stricture formation for each additional year of delay, with fibrostricturing forms rising from 19% to 92% across the same interval [44]. Our results were consistent with this continuum, as shorter DDs were accompanied by fewer fibrostricturing presentations, particularly among mixed/stricturing phenotypes, suggesting that earlier recognition may prevent progression to advanced structural remodeling. This association was further exemplified by patients diagnosed after food impaction, who showed a threefold longer delay than those without impaction (12 vs. 4 years, p = 0.009) and significantly higher total, inflammatory, and fibrostricturing EREFS scores [45]. The progressive decline in DD mirrored the improvement previously described in the EoE CONNECT network, where median delay decreased from 12.7 years before 2007 to 0.7 years after 2017, alongside a reduction in fibrostricturing phenotypes (from 41% to 16%) and lower EREFS sub‐scores [29]. This interpretation must, however, be tempered by the parallel improvement in EREFS documentation observed over the study period. The proportion of patients with sufficient EREFS data for phenotype classification rose progressively across the diagnostic periods (Table 2), from 65.8% in ≤ 2007 to 96.7% in 2023–2025, meaning that phenotype distributions in earlier periods are based on a smaller and potentially non‐representative subset. Part of the apparent shift toward inflammatory phenotypes may therefore reflect more complete and consistent documentation of subtle endoscopic features in recent years, in addition to a reduction in advanced fibrostricturing presentations only partly driven by earlier diagnosis. Collectively, these data indicate that the dissemination of clinical guidelines and increased physician awareness have translated into measurable advances in the timeliness and accuracy of EoE diagnosis across Europe.

This study had several notable strengths. It relied on the EoE CONNECT registry, a structured and high‐quality source of real‐world clinical data, representing the largest multicenter evaluation of endoscopic and biopsy practices at EoE diagnosis in Europe. The broad temporal coverage (1991–2025) enabled the assessment of genuine longitudinal trends in diagnostic behavior, while the large sample size provided robust power to evaluate segment‐specific diagnostic performance. Several limitations should be acknowledged. Participating centers likely had a greater‐than‐average interest and expertise in EoE, introducing a potential expertise bias that may limit generalizability to less specialized settings. Moreover, as the majority of centers are from Spain (83%), the trends primarily reflect Spanish, and to a lesser extent, Italian clinical practice. The classification of centers by volume (cut‐off 200 patients) is necessarily arbitrary, and all participating centers likely share a higher‐than‐average interest in EoE management, reinforcing the potential expertise bias of the analysis. The registry did not capture the exact number of biopsy fragments obtained per site, a determinant of diagnostic sensitivity [46], nor did it distinguish between the patient‐ and physician‐dependent components of DD, which have recently been recognized as distinct phases in the diagnostic pathway [47]. Histological assessment relied on peak eosinophil counts rather than systematic EoEHSS grading, limiting the evaluation of epithelial injury and remodeling beyond eosinophil density. However, this was unlikely to affect phenotype classification, which was mainly based on clinical‐endoscopic rather than histological fibrosis assessment. In addition, factors influencing biopsy sampling strategies at the individual patient level could not be assessed as this information is not systematically collected in the EoE CONNECT registry. Information on symptom onset relied solely on patient self‐report and may therefore be subject to recall bias, especially among patients with earlier symptom onset before 2010. However, these patients represented only 17.1% of the total cohort, so the potential impact of this bias may be limited. Furthermore, our study described temporal associations but cannot establish direct causal relationships between guideline publication and patient outcomes such as DD. The effect in DD and changes on phenotype distribution over time is likely a reflection of the cumulative integration of evidence‐based diagnostic practices and clinical guidelines adherence into the evolving clinical environment.

Here we offered the most comprehensive real‐world overview of how diagnostic practices for EoE have evolved across Europe. Over more than three decades, clinical practice progressively aligned with international guidelines, measured here as the adoption of standardized endoscopic reporting and multi‐segment biopsy protocols. We reported, with the largest study cohort to date, that distal containing sampling exhibits the greatest sensitivity, especially when combined with middle segments, supporting optimized biopsy strategies. Finally, the concurrent decline in DD and fibrostricturing forms indicates that earlier recognition is becoming a measurable outcome of improved clinical awareness and guideline‐based management.

## Author Contributions

Guarantor of article: Alfredo J. Lucendo, specific author contributions: Elena Grueso‐Navarro, Alfredo J. Lucendo and Emanuele Dilaghi: study design. Elena Grueso‐Navarro and Emanuele Dilaghi: database monitoring and quality assessment. Emanuele Dilaghi and Elena Grueso‐Navarro: data extraction and analysis. Emanuele Dilaghi and Elena Grueso‐Navarro: manuscript writing – original draft). Alfredo J. Lucendo: writing–review and editing: All authors collected and registered the data, and approved the final version of the article, including the authorship list.

## Conflicts of Interest

The authors declare no conflicts of interest.

## Supporting information


Supporting Information S1

## Data Availability

The data that support the findings of this study are available from the corresponding author upon reasonable request.
