# Supplementary material for: Evolution of Endoscopic and Histologic Assessment of Eosinophilic Esophagitis in Europe and Evolution of the Diagnostic Delay: Data From the EUREOS EoE CONNECT Registry
Source: United European Gastroenterol J. 2026 Aug 12;14(7):e70271. doi: 10.1002/ueg2.70271 (PMC13469753; doi:10.1002/ueg2.70271)
Supplement: Supplementary file 1 [file UEG2-14-e70271-s001.docx]

**Supplementary material**

**Evolution of endoscopic and histologic assessment of Eosinophilic Esophagitis in Europe and evolution of the diagnostic delay: Data from the EUREOS EoE CONNECT registry**

**Running title**

Endoscopy Trends and Diagnostic Delay in EoE

**Authors**

Grueso-Navarro E*^1,2,3^, Dilaghi E*^4^, Fernández-Pacheco J^5,6^, Amorena E^7^, Savarino EV^8^, Blas-Jhon L^9^, Pérez-Martínez I^10,11^, Granja-Navacerrada A^12^, Guagnozzi D^13^, Martín-Asenjo L^14^, Pellegatta G^15^, Coletta M^16^, García-Suárez C^17^, de la Riva S^18^, de la Peña-Negro L^19^, Betoré E^20^, Krarup AL^21,22^, Barrio J^23^, Votto M^24^, Bujanda L.^3,25^, Fernández-Fernández S^26^, Naves JE^27^, González-Almeida A^28^, Honrubia-López R^29,30^, Carrión S^3,31^, Oliva S^32^, Caño-Cerdán C^33^, Masiques-Mas ML^34^, Espina-Cadena S^35^, González-Cervera J^36^, Casabona-Francés S^5,6^, Nicolay-Maneru J^7^, Úbeda-Vargas V^1^, Ghisa M^8^, Romo P^9^, Donado C^37^, Benagues A^12^, Martín-Domínguez V^5,6^, Nantes-Castillejo O^7,^ Arias-González L^1,2,3^, Maniero D^8^, Moreira-González H^9^, Maray I^10,11^, Barrio-Torres J^12^, Pérez-Fernández MT^5,6^, Ostiz-Llanos M^7^, Rodríguez-Alcolado L^1,2,3,38^, Cabrera-Martínez AM^1^, Laserna-Mendieta EJ^1,2,3,5^, Santander C^3,5,6^, Lucendo AJ^1,2,3,5^

* Should be considered as first-co-authors.

**Supplementary Material and Methods**

**Study design and dataset**

Exclusion criteria were: (i) same patient assigned to different IDs, (ii) missing histology data at diagnosis, (iii) eosinophil count of <15 eos/hpf, and (iv) missing or inconsistent dates of diagnosis, endoscopy, or histology. When multiple IDs were assigned to the same patient, the record with the most complete diagnostic data was kept. Finally, 3,298 patients met the inclusion criteria.

Database monitoring and data quality assessment were performed manually at the individual patient level to ensure data coherence and completeness.

**Variables and definitions**

Hospitals were classified by caseload: centers with ≥200 registered patients were considered high-volume, and those with fewer as low-volume. DD was defined as the interval between symptom onset (by patient recall) and diagnosis, calculated as diagnosis year minus symptom onset year. The overall peak eosinophil count (PEC) corresponded to the highest value reported across all sampled esophageal segments. Biopsies were classified as pathologic if they showed a PEC ≥15 eos/hpf or recorded as *pathologic* in the registry. EoE phenotypes were defined according to EREFS, requiring sufficient information on the Rings and Strictures sub-scores: cases with an EREFS Rings sub-score ≥2 or with documented strictures were classified as mixed/stricturing, and the remaining cases as inflammatory. Patients missing Rings or Strictures subs-cores, could not be assigned a phenotype and were therefore excluded from the phenotype analysis.

**Statistical analysis**

Continuous variables were shown as mean ± standard deviation (SD) or median and interquartile range (IQR), depending on data distribution assessed with normality tests. Categorical variables were reported as absolute frequencies and percentages (n, %) and compared using the Chi-square tests. The Mann-Whitney *U* test was employed for comparisons between non-normal continuous variables.

Pairwise comparisons of sensitivity between 2-esophageal segments combinations within a cohort of patients biopsied in all three segments (proximal, middle and distal esophagus) was compared using McNemar’s test for paired binary data and *p* values adjusted for multiple comparisons using the Benjamini–Hochberg test. Temporal correlations between DD and year of symptom onset were evaluated with simple linear regression analysis. Biopsy sampling practices across six guideline-defined groups (≤2007, 2008–2011, 2012–2013, 2014–2017, 2018–2022 and 2023–2025) was compared with chi-square test, followed by binary logistic regression model to estimate odds ratios (OR) of sampling ≥2 esophageal segments in each period, with the “≤ 2007” group as reference. Sensitivity analyses were also performed using the 2008–2011, 2012–2013, and 2014–2017 periods as alternative reference categories (Supplementary Table 1) to assess the robustness of results.

**Supplementary Table 1.** Sensitivity analysis of the logistic regression of multi-segment esophageal biopsy sampling (≥2 segments) using alternative reference categories. Asterisks indicate p < 0.05

|  |  | **OR (95% CI)** | | | |
| --- | --- | --- | --- | --- | --- |
| **Period** | **N** | **Ref < 2007** | **Ref 2008–2011** | **Ref 2012–2013** | **Ref 2014–2017** |
| **<2007** | 38 | 1.00 | 0.81 (0.37-1.77) | 0.60 (0.27-1.32) | 0.45 (0.22-0.93)* |
| **2008–2011** | 136 | 1.23 (0.57-2.70) | 1.00 | 0.74 (0.43-1.27) | 0.56 (0.37-0.86)* |
| **2012–2013** | 152 | 1.66 (0.76-3.65) | 1.35 (0.79-2.31) | 1.00 | 0.76 (0.49-1.17) |
| **2014–2017** | 652 | 2.20 (1.08-4.49)* | 1.78 (1.16-2.74)* | 1.32 (0.86-2.04) | 1.00 |
| **2018–2022** | 1,570 | 3.68 (1.82-7.42)* | 2.98 (1.98-4.48)* | 2.21 (1.46-3.35)* | 1.67 (1.29-2.16)* |
| **2023–2025** | 750 | 6.90 (3.28-14.54)* | 5.59 (3.46-9.03)* | 4.15 (2.55-6.74)* | 3.14 (2.19-4.49)* |

**Supplementary Figure 1.** Linear regression of the proportion of patients undergoing biopsy sampling from ≥2 esophageal segments over time. Each data point represents one calendar year, point size is proportional to the number of patients diagnosed in that year. The dashed line represents the regression fit.

**Supplementary Figure 2.** Stacked-bar plot showing the percentage of pathologic and non-pathologic biopsies in the proximal, middle and distal esophagus in the subgroup of patients with three-segment sampling at diagnosis (n=785).
